# Supplementary figures and images for: Metabolomics Reveals Glycerophospholipids, Peptides, and Flavonoids Contributing to Breast Meat Flavor and Benefit Properties of Beijing-You Chicken
Source: Foods. 2024 Aug 15;13(16):2549. doi: 10.3390/foods13162549 (PMC11354068; doi:10.3390/foods13162549)

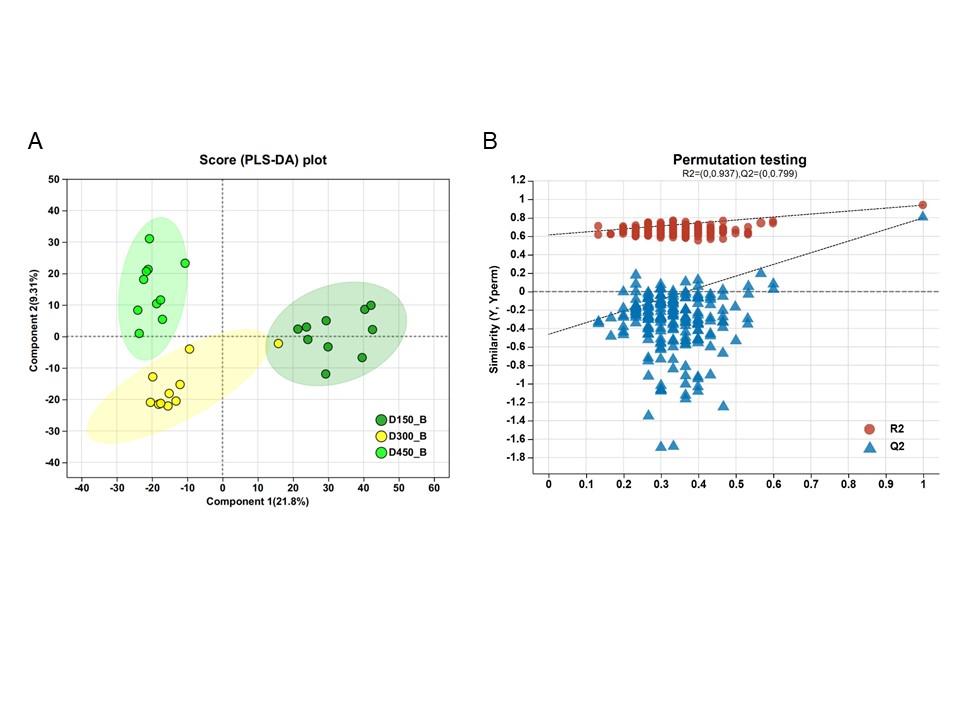

Supplement: Supplementary file 1 [file foods-13-02549-s001.zip › Figure S1 The partial least squares discriminant analysis (PLS-DA) between different age stages.jpg]

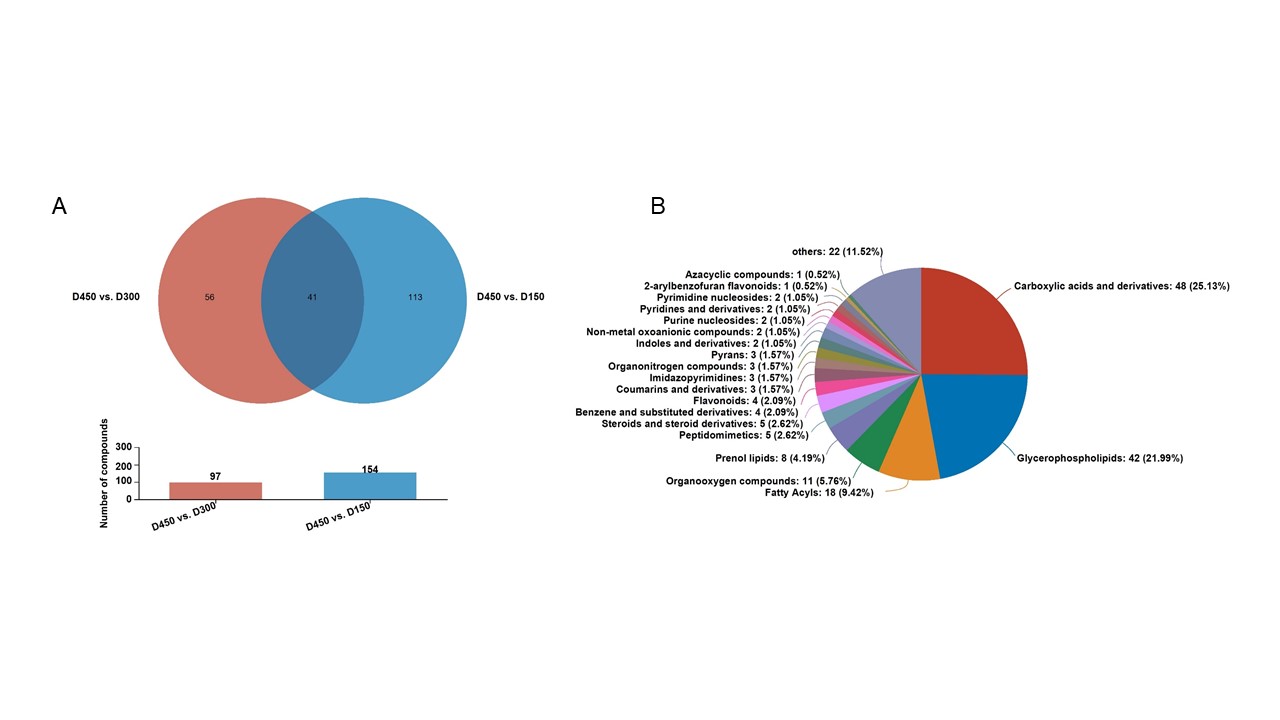

Supplement: Supplementary file 1 [file foods-13-02549-s001.zip › Figure S2 Venn diagram of differential metabolites and Categorization of differential metabolites based on human metabolome database (HMDB).jpg]
